# Poly[di-μ_3_-acetato-μ_8_-(naphthalene-1,5-disulfonato)-dilead(II)]

**DOI:** 10.1107/S160053681201834X

**Published:** 2012-04-28

**Authors:** Shan Gao, Seik Weng Ng

**Affiliations:** a Key Laboratory of Functional Inorganic Material Chemistry, Ministry of Education, Heilongjiang University, Harbin 150080, People’s Republic of China; b Department of Chemistry, University of Malaya, 50603 Kuala Lumpur, Malaysia; c Chemistry Department, Faculty of Science, King Abdulaziz University, PO Box 80203 Jeddah, Saudi Arabia

## Abstract

In the polymeric title complex, [Pb_2_(CH_3_CO_2_)_2_(C_10_H_6_O_6_S_2_)]_*n*_, the acetate anion functions in a chelating mode and both O atoms also coordinate to adjacent Pb^II^ atoms. The naphthalene-1,5-disulfonate dianion, which lies on a center of inversion, is connected to four Pb^II^ atoms. The bridging modes of the monoanion and dianion give rise to a three-dimensional coordination polymer. The Pb^II^ atom is eight-coordinate in the form of an undefined coordination polyhedron.

## Related literature

For a review of metal arene­sulfonates, see: Cai (2004 ▶). For an example of a μ_3_-chelating acetate in a lead(II) system, see: Morsali & Mahjoub (2004 ▶).
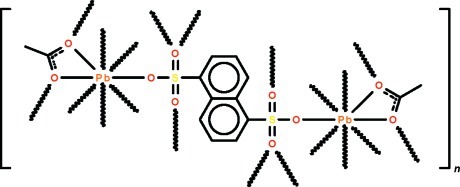


## Experimental

### 

#### Crystal data

[Pb_2_(C_2_H_3_O_2_)_2_(C_10_H_6_O_6_S_2_)]
*M* *_r_* = 818.74
Monoclinic, 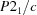

*a* = 10.442 (4) Å
*b* = 12.666 (6) Å
*c* = 6.803 (4) Å
β = 96.897 (19)°
*V* = 893.3 (7) Å^3^
*Z* = 2
Mo *K*α radiation
μ = 19.11 mm^−1^
*T* = 293 K
0.26 × 0.21 × 0.16 mm

#### Data collection

Rigaku R-AXIS RAPID IP diffractometer
Absorption correction: multi-scan (*ABSCOR*; Higashi, 1995 ▶) *T*_min_ = 0.083, *T*_max_ = 0.150
8599 measured reflections
2041 independent reflections
1922 reflections with *I* > 2σ(*I*)
*R*_int_ = 0.060

#### Refinement

*R*[*F* ^2^ > 2σ(*F* ^2^)] = 0.030
*wR*(*F* ^2^) = 0.076
*S* = 1.05
2041 reflections
129 parameters
H-atom parameters constrained
Δρ_max_ = 2.09 e Å^−3^
Δρ_min_ = −2.53 e Å^−3^

### 

Data collection: *RAPID-AUTO* (Rigaku, 1998 ▶); cell refinement: *RAPID-AUTO*; data reduction: *CrystalClear* (Rigaku/MSC, 2002 ▶); program(s) used to solve structure: *SHELXS97* (Sheldrick, 2008 ▶); program(s) used to refine structure: *SHELXL97* (Sheldrick, 2008 ▶); molecular graphics: *X-SEED* (Barbour, 2001 ▶); software used to prepare material for publication: *publCIF* (Westrip, 2010 ▶).

## Supplementary Material

Crystal structure: contains datablock(s) global, I. DOI: 10.1107/S160053681201834X/xu5522sup1.cif

Structure factors: contains datablock(s) I. DOI: 10.1107/S160053681201834X/xu5522Isup2.hkl

Additional supplementary materials:  crystallographic information; 3D view; checkCIF report

## Figures and Tables

**Fig. 1.**
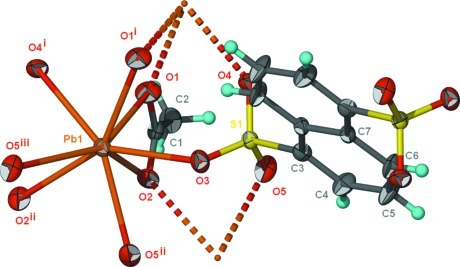
Thermal ellipsoid plot (Barbour, 2001) illustrating the geometry of PbII of polymeric Pb2(C2H3O2)2(C10H6O6S2) at the 70% probability level; hydrogen atoms are drawn as spheres of arbitrary radius.

**Fig. 2.**
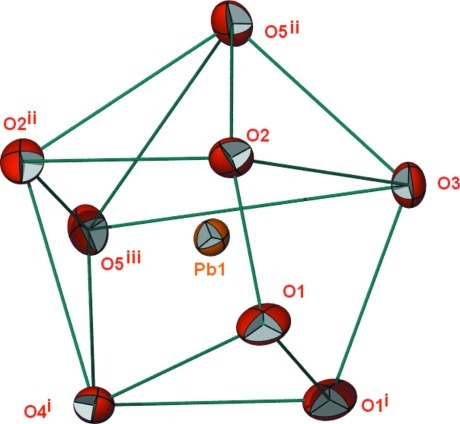
Eight-coordinate geometry of PbII.

## supplementary crystallographic information

### Comment

Metal arenesulfonates are generally crystalline compounds; in some, the metal is connected to the arenesulfonate by a covalent bond whereas in others, the arenesulfonate interacts indirectly with the metal center in an outer-sphere type of coordination (Cai, 2004). A lead(II) derivative of naphthalene-1,5-disulfonate is known in which the coordination number is high. This is attributed to the diverse binding modes of the sulfonate entity. In this example (Morsali & Mahjoub, 2004), the acetate ion engages in µ_3_-bridging. Both features are observed in polymeric Pb_2_(C_2_H_3_O_2_)_2_(C_10_H_6_O_6_S_2_) (Scheme I, Fig. 1). (The acetate anion is a part of the lead(II) acetate reactant.) The acetate ion functions in a chelating mode; both O atoms are further involved in coordinating to adjacent Pb^II^ atoms. The naphthalene-1,5-disulfonate ion lies on a center-of-inversion; the sulfonate –SO_3_ group is connected to four Pb^II^ atoms. The bridging modes of the monoanion and dianion give rise to a three-dimensional coordination polymer (Fig. 1). The Pb^II^ atom is eight-coordinate in an undefined coordination geometry (Fig. 2).

### Experimental

Equal molar amounts of lead(II) acetate (1 mmol, 327 mg) and naphthalene-1,5-disulfonic acid tetrahydrate (1 mmol, 360 mg) were dissolved in water (10 ml). The solution was filtered and then set aside for the growth of crystals. Colorless crystals separated from solution after several days.

### Refinement

Carbon- and nitrogen-bound H-atoms were placed in calculated positions (C–H 0.93–0.98 Å) and were included in the refinement in the riding model approximation, with *U*(H) set to 1.2–1.5*U*(C).

The final difference Fourier map had a peak in the vicinity of Pb1 as well as a hole in the vicinity of this atom.

### Crystal data

**Table:**

[Pb_2_(C_2_H_3_O_2_)_2_(C_10_H_6_O_6_S_2_)]	*F*(000) = 744
*M**_r_* = 818.74	*D*_x_ = 3.044 Mg m^−^^3^
Monoclinic, *P*2_1_/*c*	Mo *K*α radiation, λ = 0.71073 Å
Hall symbol: -P 2ybc	Cell parameters from 7836 reflections
*a* = 10.442 (4) Å	θ = 3.2–27.5°
*b* = 12.666 (6) Å	µ = 19.11 mm^−^^1^
*c* = 6.803 (4) Å	*T* = 293 K
β = 96.897 (19)°	Prism, brown
*V* = 893.3 (7) Å^3^	0.26 × 0.21 × 0.16 mm
*Z* = 2	

### Data collection

**Table:**

Rigaku R-AXIS RAPID IP diffractometer	2041 independent reflections
Radiation source: fine-focus sealed tube	1922 reflections with *I* > 2σ(*I*)
Graphite monochromator	*R*_int_ = 0.060
ω scan	θ_max_ = 27.5°, θ_min_ = 3.2°
Absorption correction: multi-scan (*ABSCOR*; Higashi, 1995)	*h* = −11→13
*T*_min_ = 0.083, *T*_max_ = 0.150	*k* = −15→16
8599 measured reflections	*l* = −8→8

### Refinement

**Table:**

Refinement on *F*^2^	Secondary atom site location: difference Fourier map
Least-squares matrix: full	Hydrogen site location: inferred from neighbouring sites
*R*[*F*^2^ > 2σ(*F*^2^)] = 0.030	H-atom parameters constrained
*wR*(*F*^2^) = 0.076	*w* = 1/[σ^2^(*F*_o_^2^) + (0.0383*P*)^2^ + 2.2119*P*] where *P* = (*F*_o_^2^ + 2*F*_c_^2^)/3
*S* = 1.05	(Δ/σ)_max_ = 0.001
2041 reflections	Δρ_max_ = 2.09 e Å^−^^3^
129 parameters	Δρ_min_ = −2.53 e Å^−^^3^
0 restraints	Extinction correction: *SHELXL97* (Sheldrick, 2008), Fc^*^=kFc[1+0.001xFc^2^λ^3^/sin(2θ)]^-1/4^
Primary atom site location: structure-invariant direct methods	Extinction coefficient: 0.0153 (7)

### Fractional atomic coordinates and isotropic or equivalent isotropic displacement parameters (Å^2^)

**Table:**

	*x*	*y*	*z*	*U*_iso_*/*U*_eq_	
Pb1	0.465191 (17)	0.638470 (13)	0.64777 (3)	0.01655 (12)	
S1	0.30857 (11)	0.58790 (9)	0.14316 (19)	0.0152 (3)	
O1	0.6125 (5)	0.5588 (3)	0.4215 (7)	0.0293 (10)	
O2	0.5932 (4)	0.7294 (3)	0.3948 (6)	0.0241 (9)	
O3	0.2901 (4)	0.6274 (3)	0.3396 (7)	0.0229 (9)	
O4	0.3610 (4)	0.4829 (3)	0.1445 (6)	0.0233 (8)	
O5	0.3804 (4)	0.6622 (3)	0.0349 (7)	0.0249 (9)	
C1	0.6511 (6)	0.6459 (4)	0.3576 (9)	0.0176 (11)	
C2	0.7677 (7)	0.6495 (5)	0.2497 (10)	0.0262 (13)	
H2A	0.8085	0.7172	0.2692	0.039*	
H2B	0.7424	0.6383	0.1110	0.039*	
H2C	0.8271	0.5953	0.2999	0.039*	
C3	0.1528 (5)	0.5777 (4)	0.0066 (8)	0.0171 (10)	
C4	0.1285 (6)	0.6372 (4)	−0.1604 (10)	0.0237 (13)	
H4	0.1892	0.6860	−0.1925	0.028*	
C5	0.0110 (6)	0.6250 (5)	−0.2852 (10)	0.0286 (14)	
H5	−0.0047	0.6654	−0.3997	0.034*	
C6	−0.0786 (5)	0.5550 (4)	−0.2391 (9)	0.0232 (12)	
H6	−0.1544	0.5466	−0.3245	0.028*	
C7	−0.0591 (5)	0.4946 (4)	−0.0642 (7)	0.0149 (10)	

### Atomic displacement parameters (Å^2^)

**Table:**

	*U*^11^	*U*^22^	*U*^33^	*U*^12^	*U*^13^	*U*^23^
Pb1	0.01896 (16)	0.01700 (16)	0.01406 (16)	−0.00039 (6)	0.00350 (9)	−0.00111 (6)
S1	0.0139 (5)	0.0152 (5)	0.0158 (6)	−0.0021 (4)	−0.0013 (4)	−0.0009 (4)
O1	0.042 (2)	0.0187 (18)	0.029 (3)	−0.0062 (18)	0.0096 (19)	0.0069 (17)
O2	0.027 (2)	0.0229 (18)	0.023 (2)	0.0090 (16)	0.0041 (17)	−0.0050 (16)
O3	0.019 (2)	0.0274 (19)	0.021 (2)	0.0020 (15)	−0.0027 (17)	−0.0056 (15)
O4	0.0222 (18)	0.0194 (17)	0.027 (2)	0.0057 (15)	−0.0022 (16)	−0.0030 (16)
O5	0.0185 (19)	0.0312 (19)	0.025 (2)	−0.0104 (17)	0.0004 (17)	0.0063 (18)
C1	0.020 (3)	0.018 (2)	0.014 (3)	0.0008 (18)	0.000 (2)	0.0008 (18)
C2	0.029 (3)	0.032 (3)	0.020 (3)	0.000 (2)	0.012 (3)	0.001 (2)
C3	0.013 (2)	0.017 (2)	0.021 (3)	0.0008 (19)	0.0000 (19)	−0.003 (2)
C4	0.023 (3)	0.025 (3)	0.024 (3)	−0.0028 (19)	0.004 (2)	0.010 (2)
C5	0.027 (3)	0.034 (3)	0.023 (3)	−0.007 (2)	−0.005 (3)	0.017 (2)
C6	0.020 (3)	0.029 (3)	0.018 (3)	−0.002 (2)	−0.007 (2)	0.006 (2)
C7	0.016 (2)	0.016 (2)	0.012 (3)	0.0002 (18)	0.0012 (19)	−0.0014 (18)

### Geometric parameters (Å, º)

**Table:**

Pb1—O1	2.515 (5)	O4—Pb1^i^	2.653 (4)
Pb1—O1^i^	2.652 (4)	O5—Pb1^iv^	2.754 (4)
Pb1—O2	2.574 (5)	C1—C2	1.496 (9)
Pb1—O2^ii^	2.622 (4)	C2—H2A	0.9600
Pb1—O3	2.615 (4)	C2—H2B	0.9600
Pb1—O4^i^	2.653 (4)	C2—H2C	0.9600
Pb1—O5^iii^	2.893 (5)	C3—C4	1.361 (8)
Pb1—O5^ii^	2.754 (4)	C3—C7^v^	1.429 (7)
S1—O4	1.438 (4)	C4—C5	1.414 (9)
S1—O5	1.457 (4)	C4—H4	0.9300
S1—O3	1.461 (5)	C5—C6	1.353 (8)
S1—C3	1.778 (5)	C5—H5	0.9300
O1—C1	1.269 (6)	C6—C7	1.409 (7)
O1—Pb1^i^	2.652 (4)	C6—H6	0.9300
O2—C1	1.259 (6)	C7—C3^v^	1.429 (7)
O2—Pb1^iv^	2.622 (4)	C7—C7^v^	1.431 (9)
			
O1—Pb1—O2	50.61 (14)	C1—O1—Pb1	95.9 (4)
O1—Pb1—O3	84.72 (15)	C1—O1—Pb1^i^	149.4 (4)
O2—Pb1—O3	81.78 (14)	Pb1—O1—Pb1^i^	106.87 (16)
O1—Pb1—O2^ii^	110.26 (14)	C1—O2—Pb1	93.4 (4)
O2—Pb1—O2^ii^	82.95 (9)	C1—O2—Pb1^iv^	128.9 (4)
O3—Pb1—O2^ii^	143.29 (12)	Pb1—O2—Pb1^iv^	116.65 (15)
O1—Pb1—O1^i^	73.13 (16)	S1—O3—Pb1	126.5 (2)
O2—Pb1—O1^i^	118.36 (13)	S1—O4—Pb1^i^	140.8 (2)
O3—Pb1—O1^i^	68.75 (13)	S1—O5—Pb1^iv^	128.3 (3)
O2^ii^—Pb1—O1^i^	146.84 (12)	O2—C1—O1	118.8 (6)
O1—Pb1—O4^i^	70.34 (14)	O2—C1—C2	120.8 (5)
O2—Pb1—O4^i^	103.77 (13)	O1—C1—C2	120.4 (5)
O3—Pb1—O4^i^	139.81 (11)	C1—C2—H2A	109.5
O2^ii^—Pb1—O4^i^	76.41 (13)	C1—C2—H2B	109.5
O1^i^—Pb1—O4^i^	73.99 (13)	H2A—C2—H2B	109.5
O1—Pb1—O5^ii^	113.41 (13)	C1—C2—H2C	109.5
O2—Pb1—O5^ii^	65.11 (12)	H2A—C2—H2C	109.5
O3—Pb1—O5^ii^	69.81 (12)	H2B—C2—H2C	109.5
O2^ii^—Pb1—O5^ii^	73.47 (12)	C4—C3—C7^v^	121.2 (5)
O1^i^—Pb1—O5^ii^	137.12 (15)	C4—C3—S1	117.6 (4)
O4^i^—Pb1—O5^ii^	148.89 (12)	C7^v^—C3—S1	121.1 (4)
O1—Pb1—O5^iii^	151.11 (12)	C3—C4—C5	120.2 (5)
O2—Pb1—O5^iii^	143.35 (12)	C3—C4—H4	119.9
O3—Pb1—O5^iii^	118.32 (14)	C5—C4—H4	119.9
O2^ii^—Pb1—O5^iii^	62.50 (12)	C6—C5—C4	120.5 (6)
O1^i^—Pb1—O5^iii^	98.00 (13)	C6—C5—H5	119.8
O4^i^—Pb1—O5^iii^	80.81 (13)	C4—C5—H5	119.8
O5^ii^—Pb1—O5^iii^	92.01 (12)	C5—C6—C7	121.2 (5)
O4—S1—O5	112.5 (3)	C5—C6—H6	119.4
O4—S1—O3	113.8 (2)	C7—C6—H6	119.4
O5—S1—O3	111.9 (3)	C6—C7—C3^v^	123.1 (4)
O4—S1—C3	105.1 (2)	C6—C7—C7^v^	119.3 (6)
O5—S1—C3	106.0 (2)	C3^v^—C7—C7^v^	117.6 (6)
O3—S1—C3	106.9 (3)		
			
O2—Pb1—O1—C1	6.4 (3)	O2—Pb1—O3—S1	50.2 (3)
O3—Pb1—O1—C1	90.1 (4)	O2^ii^—Pb1—O3—S1	116.6 (3)
O2^ii^—Pb1—O1—C1	−55.4 (4)	O1^i^—Pb1—O3—S1	−74.6 (3)
O1^i^—Pb1—O1—C1	159.4 (4)	O4^i^—Pb1—O3—S1	−51.4 (4)
O4^i^—Pb1—O1—C1	−122.0 (4)	O5^ii^—Pb1—O3—S1	116.6 (3)
O5^ii^—Pb1—O1—C1	24.7 (4)	O5^iii^—Pb1—O3—S1	−162.3 (2)
O5^iii^—Pb1—O1—C1	−125.0 (3)	O5—S1—O4—Pb1^i^	88.6 (4)
O2—Pb1—O1—Pb1^i^	−153.0 (2)	O3—S1—O4—Pb1^i^	−39.9 (5)
O3—Pb1—O1—Pb1^i^	−69.36 (16)	C3—S1—O4—Pb1^i^	−156.4 (4)
O2^ii^—Pb1—O1—Pb1^i^	145.14 (13)	O4—S1—O5—Pb1^iv^	−133.6 (3)
O1^i^—Pb1—O1—Pb1^i^	0.0	O3—S1—O5—Pb1^iv^	−4.0 (4)
O4^i^—Pb1—O1—Pb1^i^	78.59 (16)	C3—S1—O5—Pb1^iv^	112.1 (3)
O5^ii^—Pb1—O1—Pb1^i^	−134.68 (15)	Pb1—O2—C1—O1	11.2 (6)
O5^iii^—Pb1—O1—Pb1^i^	75.6 (3)	Pb1^iv^—O2—C1—O1	−117.4 (5)
O1—Pb1—O2—C1	−6.4 (3)	Pb1—O2—C1—C2	−165.4 (5)
O3—Pb1—O2—C1	−96.3 (3)	Pb1^iv^—O2—C1—C2	65.9 (7)
O2^ii^—Pb1—O2—C1	117.2 (3)	Pb1—O1—C1—O2	−11.5 (6)
O1^i^—Pb1—O2—C1	−35.9 (4)	Pb1^i^—O1—C1—O2	127.1 (7)
O4^i^—Pb1—O2—C1	43.1 (3)	Pb1—O1—C1—C2	165.1 (5)
O5^ii^—Pb1—O2—C1	−167.8 (4)	Pb1^i^—O1—C1—C2	−56.3 (10)
O5^iii^—Pb1—O2—C1	136.2 (3)	O4—S1—C3—C4	−120.1 (5)
O1—Pb1—O2—Pb1^iv^	130.8 (2)	O5—S1—C3—C4	−0.8 (5)
O3—Pb1—O2—Pb1^iv^	40.84 (16)	O3—S1—C3—C4	118.7 (5)
O2^ii^—Pb1—O2—Pb1^iv^	−105.7 (2)	O4—S1—C3—C7^v^	56.5 (5)
O1^i^—Pb1—O2—Pb1^iv^	101.22 (19)	O5—S1—C3—C7^v^	175.8 (4)
O4^i^—Pb1—O2—Pb1^iv^	−179.76 (14)	O3—S1—C3—C7^v^	−64.7 (5)
O5^ii^—Pb1—O2—Pb1^iv^	−30.66 (15)	C7^v^—C3—C4—C5	−2.7 (9)
O5^iii^—Pb1—O2—Pb1^iv^	−86.7 (2)	S1—C3—C4—C5	173.9 (5)
O4—S1—O3—Pb1	54.8 (3)	C3—C4—C5—C6	0.6 (10)
O5—S1—O3—Pb1	−74.1 (3)	C4—C5—C6—C7	1.7 (10)
C3—S1—O3—Pb1	170.2 (2)	C5—C6—C7—C3^v^	177.9 (6)
O1—Pb1—O3—S1	−0.7 (3)	C5—C6—C7—C7^v^	−1.8 (10)

Symmetry codes: (i) −*x*+1, −*y*+1, −*z*+1; (ii) *x*, −*y*+3/2, *z*+1/2; (iii) *x*, *y*, *z*+1; (iv) *x*, −*y*+3/2, *z*−1/2; (v) −*x*, −*y*+1, −*z*.
